# Alveolar macrophage-derived microvesicles mediate acute lung injury

**DOI:** 10.1136/thoraxjnl-2015-208032

**Published:** 2016-06-10

**Authors:** Sanooj Soni, Michael R Wilson, Kieran P O'Dea, Mariko Yoshida, Umar Katbeh, Samantha J Woods, Masao Takata

**Affiliations:** Section of Anaesthetics, Pain Medicine and Intensive Care, Faculty of Medicine, Imperial College London, Chelsea and Westminster Hospital, London, UK

**Keywords:** ARDS, Macrophage Biology, Cytokine Biology

## Abstract

**Background:**

Microvesicles (MVs) are important mediators of intercellular communication, packaging a variety of molecular cargo. They have been implicated in the pathophysiology of various inflammatory diseases; yet, their role in acute lung injury (ALI) remains unknown.

**Objectives:**

We aimed to identify the biological activity and functional role of intra-alveolar MVs in ALI.

**Methods:**

Lipopolysaccharide (LPS) was instilled intratracheally into C57BL/6 mice, and MV populations in bronchoalveolar lavage fluid (BALF) were evaluated. BALF MVs were isolated 1 hour post LPS, assessed for cytokine content and incubated with murine lung epithelial (MLE-12) cells. In separate experiments, primary alveolar macrophage-derived MVs were incubated with MLE-12 cells or instilled intratracheally into mice.

**Results:**

Alveolar macrophages and epithelial cells rapidly released MVs into the alveoli following LPS. At 1 hour, the dominant population was alveolar macrophage-derived, and these MVs carried substantive amounts of tumour necrosis factor (TNF) but minimal amounts of IL-1β/IL-6. Incubation of these mixed MVs with MLE-12 cells induced epithelial intercellular adhesion molecule-1 (ICAM-1) expression and keratinocyte-derived cytokine release compared with MVs from untreated mice (p<0.001). MVs released in vitro from LPS-primed alveolar macrophages caused similar increases in MLE-12 ICAM-1 expression, which was mediated by TNF. When instilled intratracheally into mice, these MVs induced increases in BALF neutrophils, protein and epithelial cell ICAM-1 expression (p<0.05).

**Conclusions:**

We demonstrate, for the first time, the sequential production of MVs from different intra-alveolar precursor cells during the early phase of ALI. Our findings suggest that alveolar macrophage-derived MVs, which carry biologically active TNF, may play an important role in initiating ALI.

**Key messages**

**What is the key question?**

- What is the role of microvesicles in acute lung injury?

**What is the bottom line?**

- We demonstrate for the first time that alveolar macrophage-derived microvesicles are rapidly released in early acute lung injury and that these microvesicles are potent initiators of inflammation, mediated by packaged molecular cargo, particularly TNF.

**Why read on?**

- This is the first report to demonstrate the sequential production of microvesicles from different precursor cells within the alveoli during the early phase of acute lung injury, highlighting a role for alveolar macrophage-derived microvesicles as key components in the pathophysiology of acute lung injury and potentially novel therapeutic targets.

## Introduction

Acute lung injury (ALI) and its clinical presentation, acute respiratory distress syndrome (ARDS), have unacceptably high mortality, surpassing 40% in those with the severest form of the disease.^1^ ^2^ Patients that survive often have significant morbidity and endure extensive physical and psychological disability, consuming considerable healthcare resources. Despite intense research and numerous preclinical studies identifying various biological mediators as potential therapeutic targets, there are no specific therapies for ARDS, and treatment is limited to supportive care and lung-protective ventilation.^3^ As such, there remains an urgent, unmet need for a redirection in ARDS research elucidating new insights into its complex pathophysiology.

Microvesicles (MVs) are part of a group of membrane-circumscribed extracellular particles, which also include exosomes and apoptotic bodies.^4^ MVs range between 0.1 and 1 μm in size and are derived from eukaryotic cells following direct damage, apoptosis or activation. They contain elements retained from their precursor cells and carry a variety of molecular cargo, including proteins, receptors and nucleic acids^5–7^ over a distance to remote cells. Consequently, MVs provide an alternative yet crucial pathway for intercellular communication in addition to soluble mediators and direct cell-to-cell contact,^6^ ^8^ and have thus been implicated in the pathophysiology of various inflammatory diseases.^9–11^ Despite this emerging interest, there is a paucity of literature investigating the role of MVs in ARDS (or any other respiratory pathology), and in particular their bioactivity within the intra-alveolar space. The presence of MVs of various cell origins including endothelial, epithelial or platelet-derived MVs within bronchoalveolar lavage fluid (BALF) has been confirmed in patients with established ARDS^12^ ^13^ and a preclinical model of ventilator-induced lung injury.^14^ However, whether these are merely present as markers of cellular damage or key components of the pathophysiological process in ARDS is yet to be determined. Moreover, although alveolar macrophages play a central role in the evolution of lung inflammation in ALI,^15^ ^16^ they have not been previously investigated as a significant source of MVs within the alveoli.

We hypothesised that MVs released within the alveolar space play an important role in initiating lung inflammation during the early phase of ALI. Using an in vivo lipopolysaccharide (LPS) model of ALI, we were able to demonstrate that MVs are rapidly released by intra-alveolar cells and that alveolar macrophages are the dominant source in the initial stages. Using in vitro and in vivo models, we also established that alveolar macrophages release tumour necrosis factor (TNF)-containing MVs which trigger ALI.

## Materials and methods

All protocols were approved by the Ethical Review Board of Imperial College London, carried out in accordance with the Animals (Scientific Procedures) Act 1986 UK and reported in compliance with the ARRIVE guidelines. Seventy-one male C57BL/6 mice (Charles River, Margate, UK) aged between 10–14 weeks were used. Additional details for all methods are provided in the online supplementary material.

10.1136/thoraxjnl-2015-208032.supp1 Supplementary data

### MV kinetics in LPS-induced ALI in vivo

Mice were anaesthetised (intraperitoneal ketamine 90 mg/kg; xylazine 10 mg/kg), and 20 µg ‘ultrapure’ LPS (*E. coli* O111:B4) in 50 µL was instilled intratracheally (i.t.).^17^ ^18^ After 1 or 4 hours, animals were euthanised and tracheostomised. BALF samples were recovered, incubated with fluorescence-conjugated antibodies against CD11c/F4/80 (to identify alveolar macrophage-derived MVs), EpCAM (epithelial cell-derived MVs) or CD11b/Ly6G (neutrophil-derived MVs), and then analysed by flow cytometry. MVs were defined as events that were under 1 µm in size (estimated using sizing beads) and positive for specific surface markers, and their absolute counts were assessed using counting beads. The identity of MVs was validated through their sensitivity to 0.1% Triton X-100 detergent, which solubilises lipid membranes, and events resistant to the detergent were subtracted from the ‘total’ event counts to produce a final MV number.^19^ Counts were compared with those obtained from untreated control mice.

### Biological activities of BALF MVs

BALF samples, harvested from mice 1 hour after i.t. LPS or untreated mice, were centrifuged (200 g, 5 min, 4°C) to remove cells and larger particles, and the resulting supernatant was centrifuged again at high speed (20 000 g, 30 min, 4°C)^20^ ^21^ to isolate MVs. MV samples were then treated with 1% Tween detergent to disrupt their membrane and allow assessment of proinflammatory cargo using ELISA kits for TNF, IL-1β and IL-6. MV samples were also incubated with fluorescence-labelled anti-TNF antibody, and surface TNF expression on alveolar macrophage-derived (CD11c^+^) MVs was evaluated by flow cytometry.

**Figure 1:**
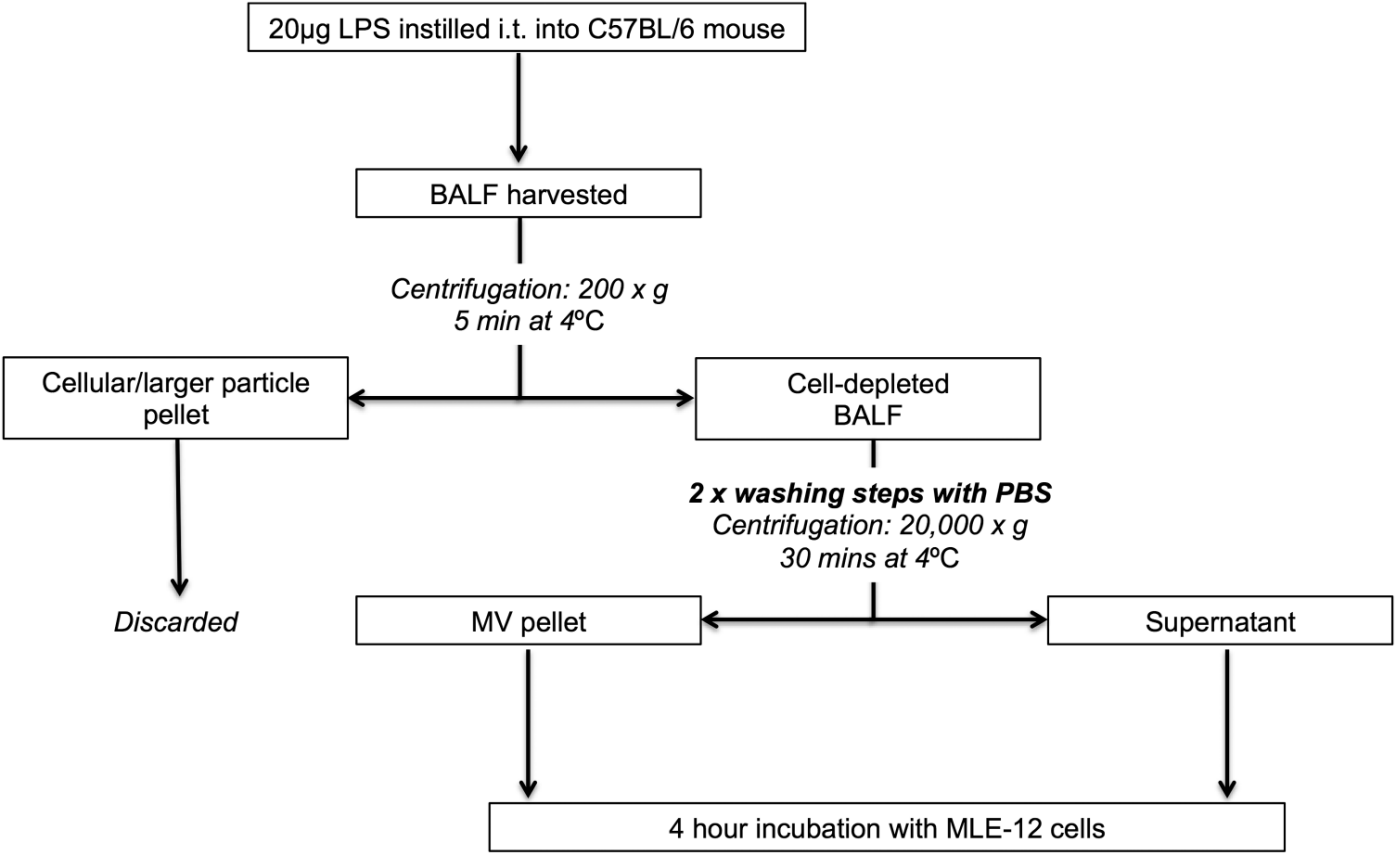
Protocol to harvest and assess the bioactivity of microvesicles (MVs) generated in vivo. 20 µg Lipopolysaccharide (LPS) was instilled i.t. into mice. Bronchoalveolar lavage fluid (BALF) was harvested 1 hour after the LPS challenge (or from untreated mice) using 700 µL normal saline and then centrifuged to remove cells and larger particles (200 g, 5 min at 4°C). Cell/larger particle pellet was discarded, and the supernatant was centrifuged at high speed (20 000 g, 30 min at 4°C) to isolate MVs, which were washed twice in PBS. These MV pellets were incubated with murine lung epithelial (MLE-12) cells for 4 hours in 24-well plates. Following the 4 hour incubation, keratinocyte-derived cytokine (KC) in cellular supernatant was determined using ELISA kits, and surface ICAM-1 expression of MLE-12 cells was assessed via flow cytometry. Post-wash supernatant, which was separated from the MV pellet after the final washing and centrifugation step, was also incubated with MLE-12 cells to differentiate MV-associated effects from those induced by soluble factors, for example, LPS or soluble BALF inflammatory mediators. ICAM-1, intercellular adhesion molecule-1; PBS, phosphate-buffered saline.

In separate experiments, MV samples isolated from BALF either 1 hour post LPS or from untreated mice were washed twice (to minimise effects of any remnant-soluble mediators) and incubated with murine lung epithelial cells (MLE-12) to assess their biological activity (figure 1). After 4 hours incubation (37°C), culture media were collected for keratinocyte-derived cytokine (KC) measurement (by ELISA), and MLE-12 cells were assessed for surface intercellular adhesion molecule-1 (ICAM-1) expression by flow cytometry.^22^ ^23^

#### Biological activities of MVs generated in vitro from alveolar macrophages

Primary alveolar macrophages were harvested by BALF from untreated mice. They were either primed for 1 hour with 1 µg/mL of LPS (37°C) to attain a proinflammatory phenotype^24^ or incubated with phosphate-buffered saline (PBS) alone, and then stimulated with 6 mM ATP,^25^ 1 mM ecto-ATPase inhibitor (ARL67156) and 40 µM calcium ionophore (A23187)^26^ for 2 hours to generate MVs of either ‘inflammatory’ or ‘non-inflammatory’ phenotype in vitro. MVs and MV-depleted supernatants were separated by centrifugation (see online supplementary information) and incubated with MLE-12 cells for 4 hours, with and without 10 µg/mL polyclonal TNF-neutralising antibody. ICAM-1 expression on MLE-12 cells was evaluated by flow cytometry.

### In vivo activities of alveolar macrophage-derived MVs

**Figure 2:**
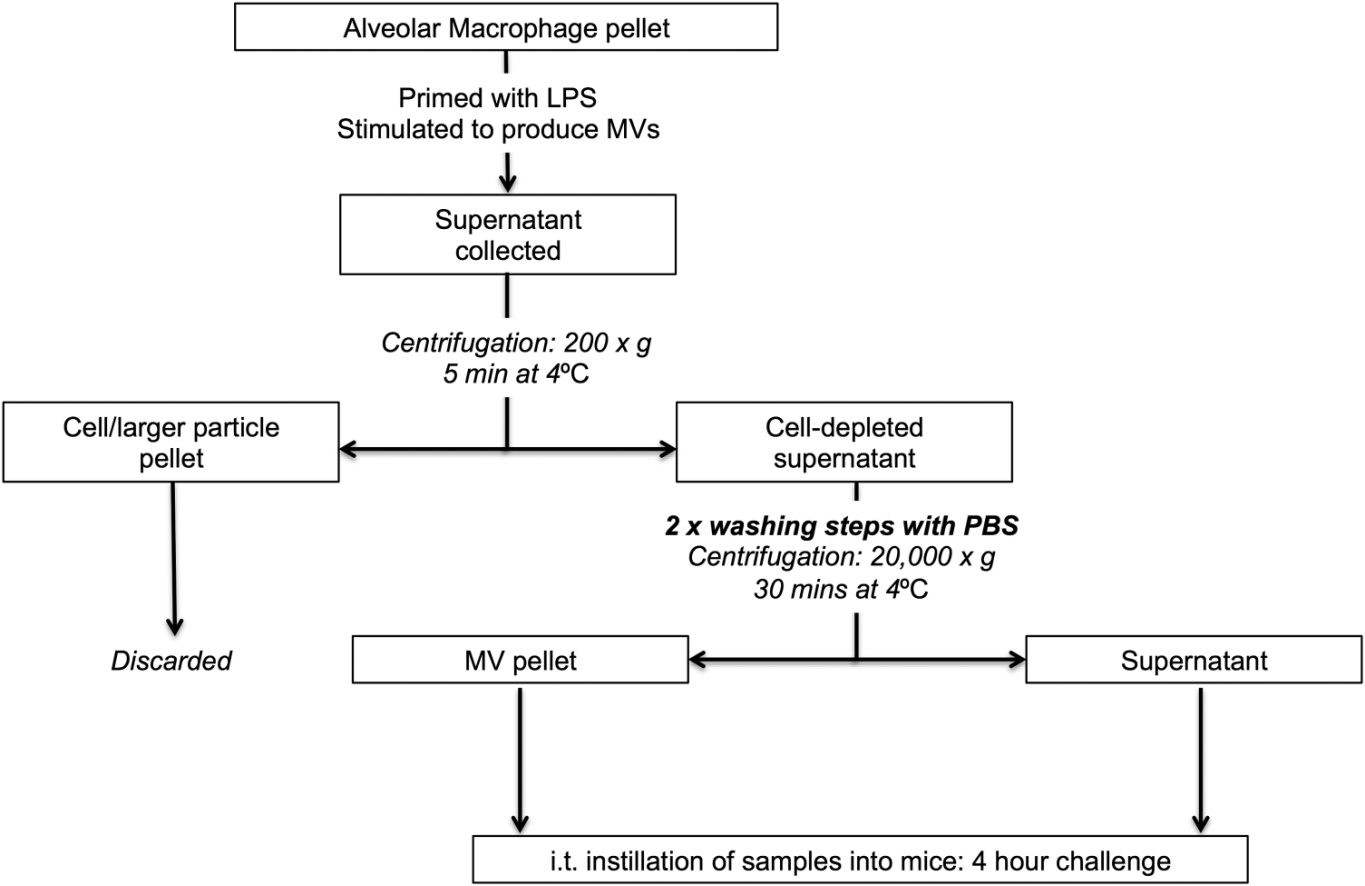
I.t. instillation of in vitro-produced primary alveolar macrophage-derived microvesicles (MVs). Harvested primary alveolar macrophages were placed in 24-well plates, primed for 1 hour with 1 µg/mL of lipopolysaccharide (LPS) to attain a proinflammatory phenotype and then stimulated with 6 mM ATP, 40 µM calcium ionophore (A23187) and 1 mM ecto-ATPase inhibitor (ARL67156) for 2 hours to generate MVs. Supernatants were subsequently collected and centrifuged to remove cells/cell debris (200 g for 5 min at 4°C). Cell-depleted supernatants were centrifuged at high speed (20 000 g for 30 min at 4°C) to isolate MVs, which were washed twice. These primary alveolar macrophage-derived MVs were then instilled i.t. into untreated, naïve mice. Post-wash supernatants, separated after the final washing step, were also collected for i.t. instillation as controls to account for any soluble factors from the MV generation that remained despite washing steps. Following a 4 hour challenge, four parameters of acute respiratory distress syndrome (ARDS) were examined: bronchoalveolar lavage fluid (BALF) protein, BALF keratinocyte-derived cytokine (KC), BALF neutrophil count and surface ICAM-1 expression on epithelial cells. ICAM-1, intercellular adhesion molecule-1.

In vitro-generated ‘inflammatory’ alveolar macrophage-derived MVs were washed to remove any remaining stimulatory factors/soluble mediators (figure 2). Washed MVs or post-wash supernatant were then instilled i.t. into the lungs of randomly selected mice, by an investigator blinded to the treatment groups. Four hours after instillation, mice were euthanised, and BALF samples were analysed for neutrophil counts, total protein levels using Bradford assay and KC levels by ELISA. Lungs were removed, and single cell suspensions were prepared for flow cytometric evaluation of ICAM-1 expression on epithelial cells using previously described methods^17^ (see online supplementary material).

### Statistical analysis

Shapiro-Wilk normality tests were carried out, and wherever possible, non-parametric data were transformed. Comparisons between two groups were performed using either paired t tests or Wilcoxon rank sum test. Where three or more groups were present, analysis of variance (ANOVA) with Tukey's HSD or Kruskal-Wallis with Dunn's test was used. Parametric data are presented as mean with SD (untransformed data) or 95% CI (transformed data), whereas non-parametric data are displayed as median with IQR. Statistical significance was defined as p<0.05, and data were analysed and graphed using IBM SPSS and Prism software.

## Results

### MVs are sequentially released from different intra-alveolar cells during ALI

**Figure 3:**
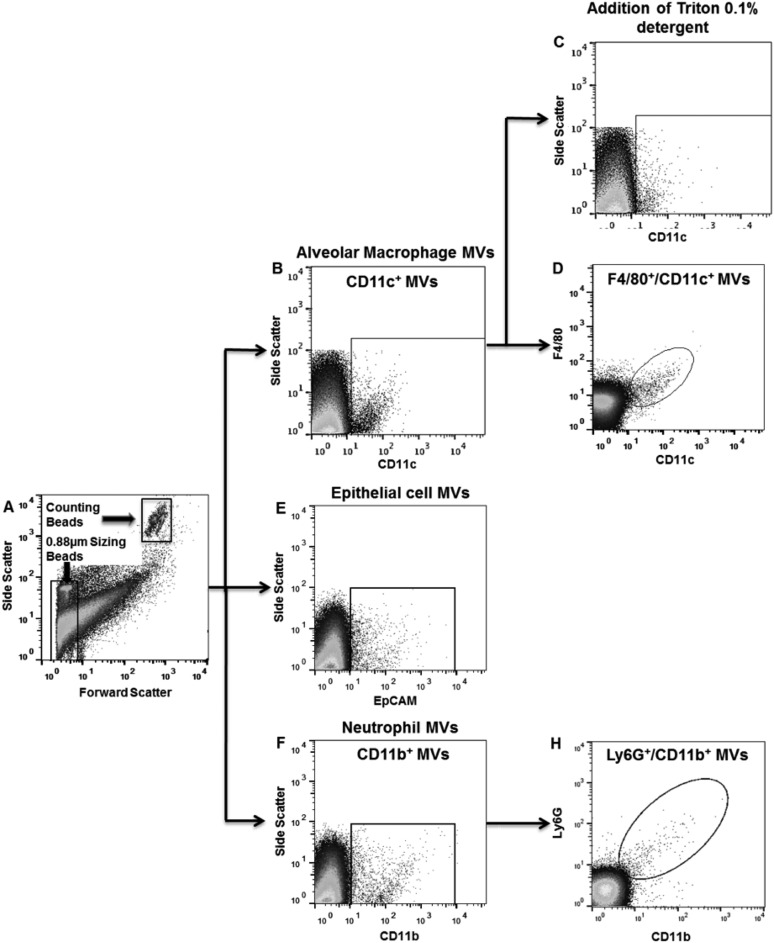
Representative plots demonstrating microvesicle (MV) characterisation in bronchoalveolar lavage fluid (BALF) in mice challenged with i.t. lipopolysaccharide (LPS) for either 1 or 4 hours. (A) Flow cytometry plot demonstrating the gating strategy to identify events under 1 µm in size using 0.88 µm sizing beads. Note that the counting beads shown in the figure have a size of 6 µm. (B) Gating strategy 1 hour post LPS demonstrating a population of CD11c^+^ events that were under 1 µm in size and thus ascribed as alveolar macrophage-derived MVs. There is a clear differentiation of CD11c^+^ events from CD11c^−^ material and the background noise (optical and electrical noise) generated by the flow cytometer. (C) Addition of 0.1% triton detergent causes disintegration of the CD11c^+^ population, as vesicle membranes are solubilised by triton, further confirming their nature as MVs. CD11c^+^ events resistant to detergent (potentially antibody complexes) were subtracted from final MV counts. (D) CD11c^+^ events were also found to be F4/80-positive, confirming their interpretation as alveolar macrophage-derived MVs. (E) Gating strategy to determine number of epithelial cell MVs (EpCAM^+^ and under 1 µm in size) 1 hour post LPS. (F) CD11b+ events were apparent after 4 hours. (G) These CD11b+ cells were also positive for Ly6G, and therefore classified as neutrophil-derived MVs.

**Figure 4:**
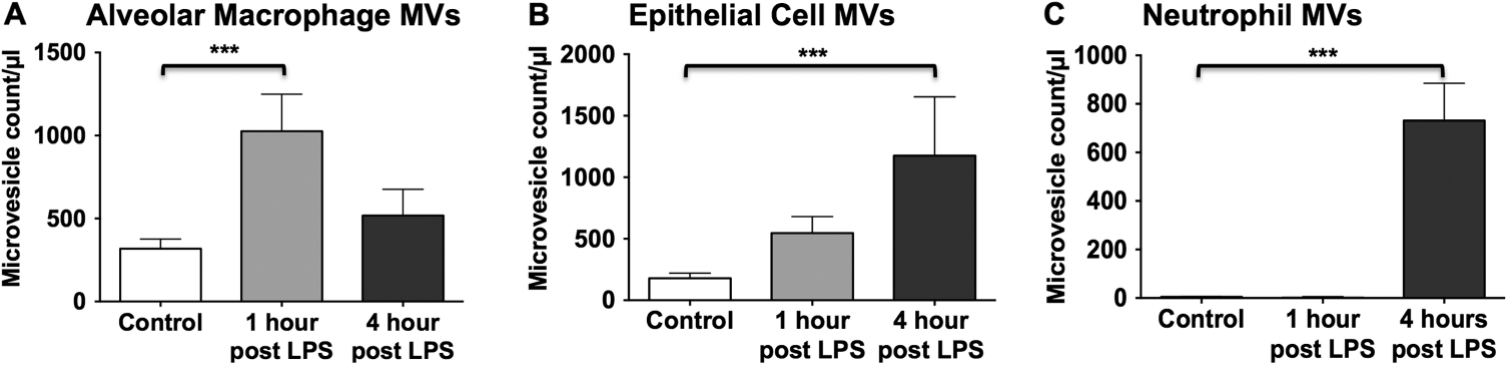
Sequential release of microvesicles (MVs) from different intra-alveolar precursor cells. MV counts are expressed as the number of detergent-sensitive (ie, total minus detergent-resistant) events <1 µm in size expressing parent cell antigens per µL of bronchoalveolar lavage fluid (BALF). (A) Histogram demonstrating the numbers of alveolar macrophage-derived MVs in BALF from untreated control mice, and 1 hour and 4 hours after i.t. lipopolysaccharide (LPS). Numbers peaked at 1 hour and fell thereafter. (B) Epithelial cell MVs continued to increase with time from injury, reaching significance after 4 hours. (C) Neutrophil-derived MVs were undetectable until 4 hours after LPS instillation. N=5–8 ***p<0.001. Data were analysed using ANOVA with Tukey's HSD and displayed as mean+SD. ANOVA, analysis of variance.

To reliably identify MVs by flow cytometry in BALF, we defined three separate criteria that had to be satisfied: (1) size less than 1 µm; (2) positive for specific precursor cell markers and (3) sensitivity to 0.1% Triton X-100 detergent, as illustrated in figure 3. Using this methodology, the dynamics of MV release from alveolar macrophages, epithelial cells and neutrophils within the alveolar space were determined during the early phase of LPS-induced ALI in mice (figure 4). Under control non-stimulated conditions, there were already populations of MVs originating from alveolar macrophages and epithelial cells present. Following LPS challenge, the number of MVs in BALF increased rapidly. MVs originating from alveolar macrophages were apparently the dominant MV population at 1 hour post LPS (1026±223/µL), and decreased thereafter. Epithelial cell-derived MVs continued to increase from the time of challenge and were the largest MV population 4 hours post LPS (1177±477/µL). Neutrophil-derived MVs were detected only in minimal numbers under control conditions and at 1 hour after LPS, but showed a large increase at 4 hours (731±153/µL), which correlates with the anticipated kinetics of neutrophil migration into the alveoli.^27^

### In vivo-produced BALF MVs have significant ‘proinflammatory’ activity

**Figure 5:**
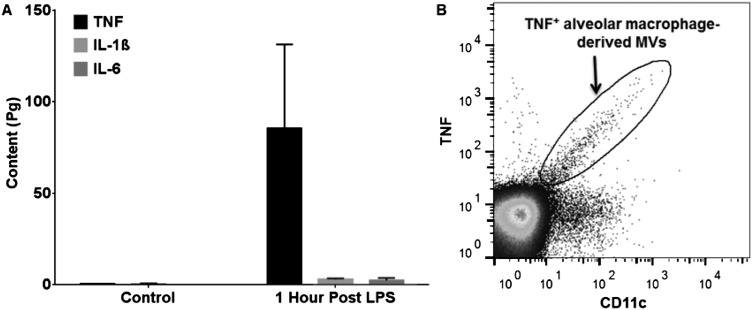
TNF, IL-1β and IL-6 content of in vivo-derived microvesicles (MVs). (A) MV pellets were isolated from bronchoalveolar lavage fluid (BALF) harvested from either untreated control mice or 1 hour post lipopolysaccharide (LPS). MVs were then treated with 1% Tween detergent, which allowed measurement of surface and intravesicular cytokines via ELISA kits. No discernable cytokines were measured in MV pellets taken from control mice. MVs obtained from those mice treated with LPS contained a substantial amount of TNF, but minimal amounts of IL-1β and IL-6. Data are displayed as mean+SD, N=5. (B) MVs extracted from BALF taken 1 hour post i.t. LPS. As seen, there is a proportion of alveolar macrophage-derived MVs which stained positive for TNF, highlighting their proinflammatory phenotype.

We then evaluated the biological activity of BALF MVs to ascertain whether MVs released in response to LPS contain enhanced proinflammatory signalling capacity rather than merely being markers of lung inflammation. We first investigated MV-associated content of TNF, IL-1β and IL-6 as indices of their proinflammatory activity, because these cytokines are important mediators of ALI^18^ ^28–30^ and are known to be expressed within the alveoli in the early phase of ALI. MVs were isolated from BALF samples taken from mice at 1 hour after i.t. LPS and analysed by ELISA (figure 5A). A significant amount of TNF was present, while minimal levels of IL-1β and IL-6 were found within these MV pellets. No discernable levels of these cytokines were found in the MV pellets taken from BALF of untreated control mice. Since alveolar macrophages are the primary source of TNF within the alveolar space^28^ and were found to be the dominant MV source at this time point of LPS-induced ALI, we determined whether MVs specifically of alveolar macrophage origin express TNF. Flow cytometric analysis clearly demonstrates that a substantial proportion (mean 22.3%, n=6) of alveolar macrophage-derived MVs were surface TNF-positive, confirming them as displaying a proinflammatory phenotype (figure 5B).

Next, we carried out experiments to evaluate whether these MVs in BALF from LPS-treated mice were able to elicit inflammatory responses in recipient cells in vitro. Specifically, we isolated MVs from mice 1 hour post LPS or control untreated mice, and incubated them with MLE-12 cells. As markers of cell activation, we studied surface ICAM-1 expression and KC production (a murine IL-8 homologue), both of which are predominantly regulated by TNF in MLE-12 cells.^31^ ^32^ In order to differentiate MV-associated effects from those induced by any potential contaminating factors that remained from LPS-treated mice despite the extra washing steps, additional controls included: (1) post-wash, MV-depleted supernatant; (2) a low dose of LPS (11 ng/mL in PBS), a theoretically calculated maximum LPS dose that may have been still retained in the final MV pellet samples; (3) a high dose of LPS (100 µg/mL in PBS), to evaluate responses in the event that MVs specifically carried and concentrated LPS on their surface and (4) PBS.

**Figure 6:**
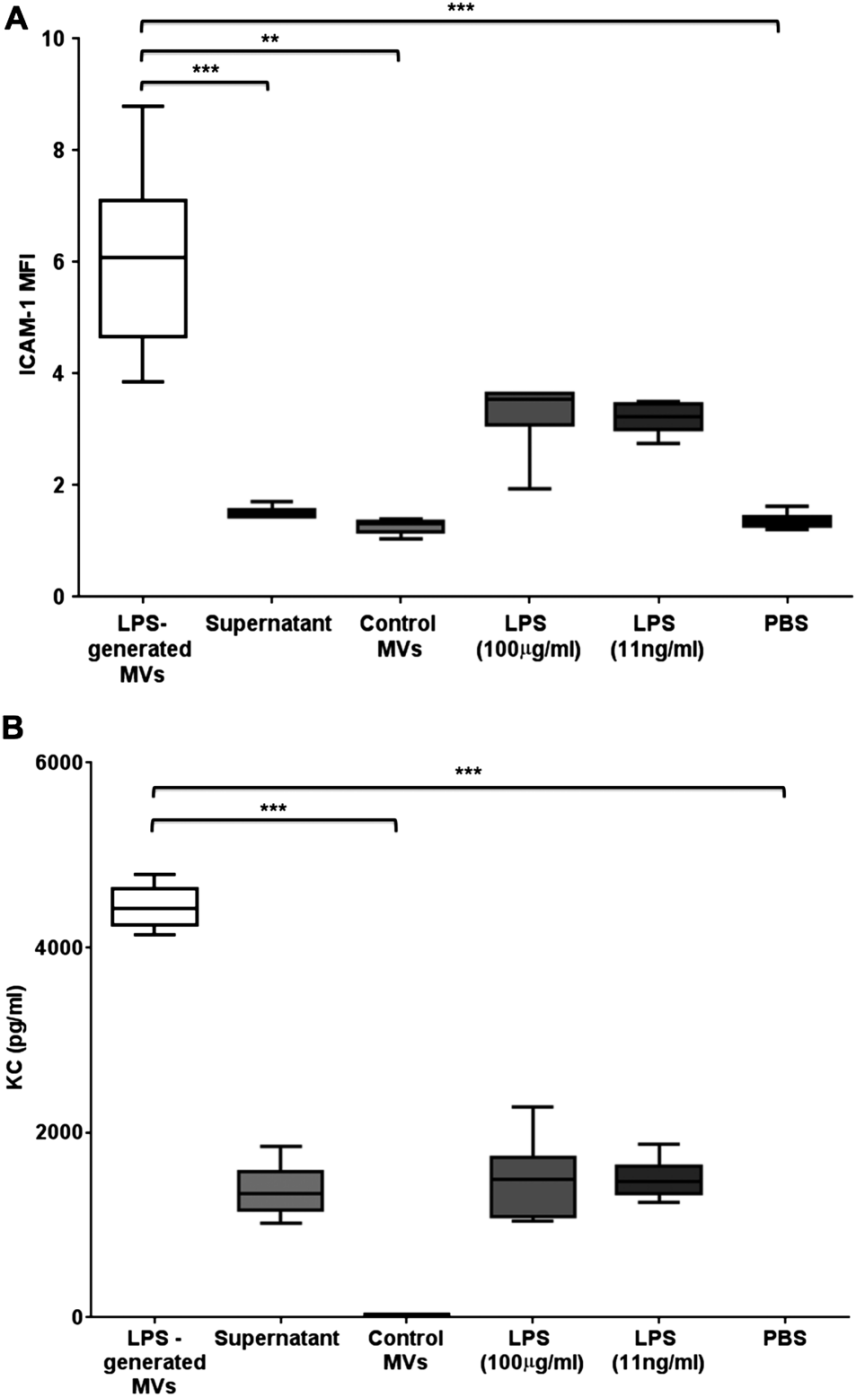
Proinflammatory activity of in vivo-derived microvesicles (MVs). (A) The effects of different treatments on ICAM-1 expression on MLE-12 cells (expressed as mean fluorescent intensity (MFI)). MVs obtained from bronchoalveolar lavage fluid (BALF) of lipopolysaccharide (LPS)-treated mice caused the largest increase when incubated with MLE-12 cells for 4 hours. This effect was significantly greater than that caused by either control MVs (obtained from BALF of untreated mice), MV-depleted supernatant or PBS. There were slight increases in MLE-12 ICAM-1 expression following high-dose and low-dose LPS, but these were not significantly different from PBS and markedly less compared with the effect of MVs. (B) The effects of different treatments on keratinocyte-derived cytokine (KC) production by MLE-12 cells. Again, MVs obtained from BALF of LPS-treated mice caused the largest production of KC, which was significantly greater than control MVs and PBS, and substantially greater than the MV-depleted supernatant and LPS. N=5–6 **p<0.01, ***p<0.001. Data were analysed with Kruskal-Wallis with Dunn’s, and displayed by box–whisker plots showing the median, IQR and minimum/maximum values. ICAM-1, intercellular adhesion molecule-1; PBS, phosphate-buffered saline.

Addition of MVs from LPS-treated mice induced significant increases in ICAM-1 expression and KC release, compared with MLE cells exposed to control MVs (figure 6). KC production by MLE cells incubated with post-wash supernatant was higher than that following PBS and comparable to that with LPS (either low or high doses), suggesting some retention of soluble factors arising from the in vivo instillation of LPS despite the washing steps. However, MVs clearly caused much greater upregulation of both ICAM-1 and KC, even compared with a high dose of LPS, highlighting that MVs released in response to i.t. LPS have substantial proinflammatory activity.

### In vitro-generated alveolar macrophage-derived MVs activate epithelial cells

**Figure 7:**
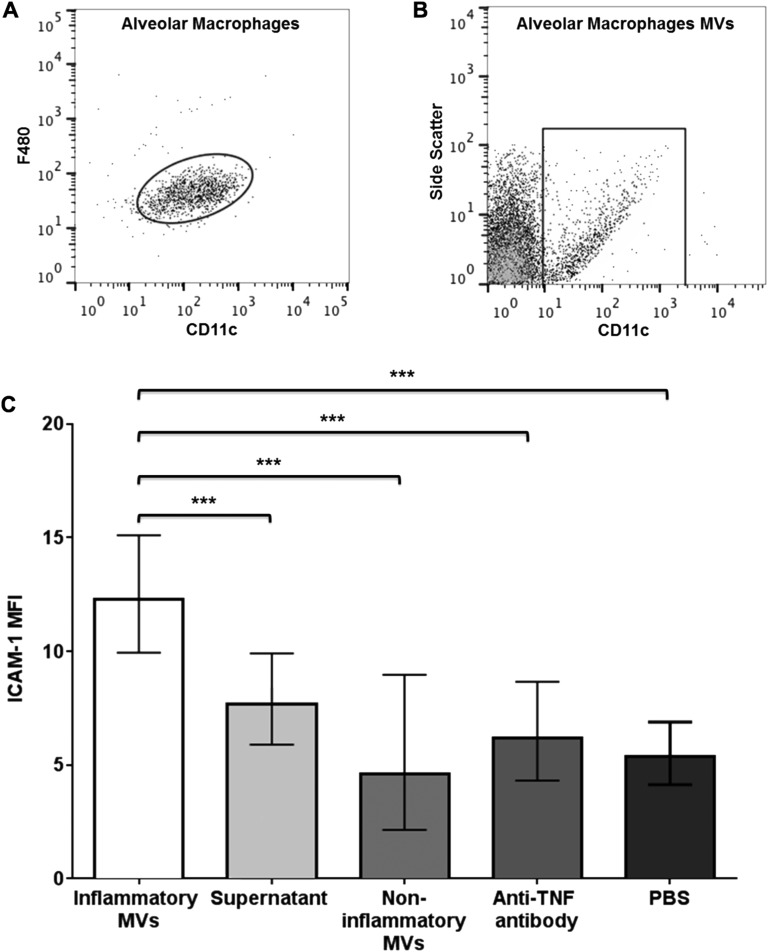
Proinflammatory activity of in vitro-generated primary alveolar macrophage-derived microvesicles (MVs). (A) Flow cytometry plot demonstrating alveolar macrophages (CD11c^+^ and F4/80^+^) extracted from bronchoalveolar lavage fluid (BALF). As expected, cells recovered from BALF were almost entirely alveolar macrophages. Therefore, MVs produced by this in vitro system were virtually exclusively derived from alveolar macrophages. (B) These macrophages were either primed with lipopolysaccharide (LPS) or incubated in PBS for 1 hour and then stimulated with ATP, A23187 and ARL67156 in vitro to produce either ‘inflammatory’ or ‘non-inflammatory’ MVs. These were identified as CD11c^+^ events that were under 1 µm in size, in keeping with the MVs originating from alveolar macrophage identified in the in vivo LPS model. (C) Alveolar macrophage-derived ‘inflammatory’ MVs were incubated with MLE-12 cells for 4 hours, which produced a significant increase in ICAM-1 expression when compared with non-inflammatory MVs, the associated supernatant fraction (obtained from the same MV-generating preparations) and PBS. Anti-TNF antibody applied to MLE-12 cells prior to MV treatment abolished this increase in ICAM-1, confirming MVs as having clear bioactivity mediated by TNF. N=3–5, ***p<0.001. Data were analysed by ANOVA with Tukey's HSD and displayed as back-transformed mean with 95% CIs (lower, upper bounds) for transformed data. ANOVA, analysis of variance; ICAM-1, intercellular adhesion molecule-1; PBS, phosphate-buffered saline.

The data described above convincingly demonstrate that MVs of mixed origin, released within the alveoli during LPS-induced ALI, can induce inflammatory activation of epithelial cells. The results also indicate that MVs originating from alveolar macrophages displayed inflammatory phenotype in response to i.t. LPS, and thus have the potential to play a role in this process. Therefore, we explored the role of MVs generated in vitro specifically from alveolar macrophages in mediating inflammatory cell activation. Primary alveolar macrophages were harvested by BALF from untreated mice, primed with LPS and then stimulated with ATP, A23187 and ARL67156 to produce inflammatory MVs (figure 7). These MVs were isolated by high-speed centrifugation, and then incubated with MLE-12 cells for 4 hours after which surface ICAM-1 on epithelial cells was examined. Alveolar macrophage-derived ‘inflammatory’ MVs caused a significant increase in ICAM-1, compared with: (1) its associated supernatant fraction; (2) ‘non-inflammatory’ MVs, which were produced from non-LPS primed (incubated with PBS alone) macrophages and (3) PBS (figure 7C). This replicates the inflammatory activity observed for the in vivo-produced BALF MVs, and is consistent with previous literature demonstrating epithelial cell activation following exposure to monocyte-derived MVs for 18 hours,^33^ although our results showed clear activation at a much shorter incubation period. Furthermore, the addition of anti-TNF antibodies to the culture system effectively abolished the ICAM-1 upregulation, providing distinct evidence that mediators carried by MVs, particularly TNF, are able to activate epithelial cells.

### In vitro-generated alveolar macrophage-derived MVs initiate ALI in vivo

**Figure 8:**
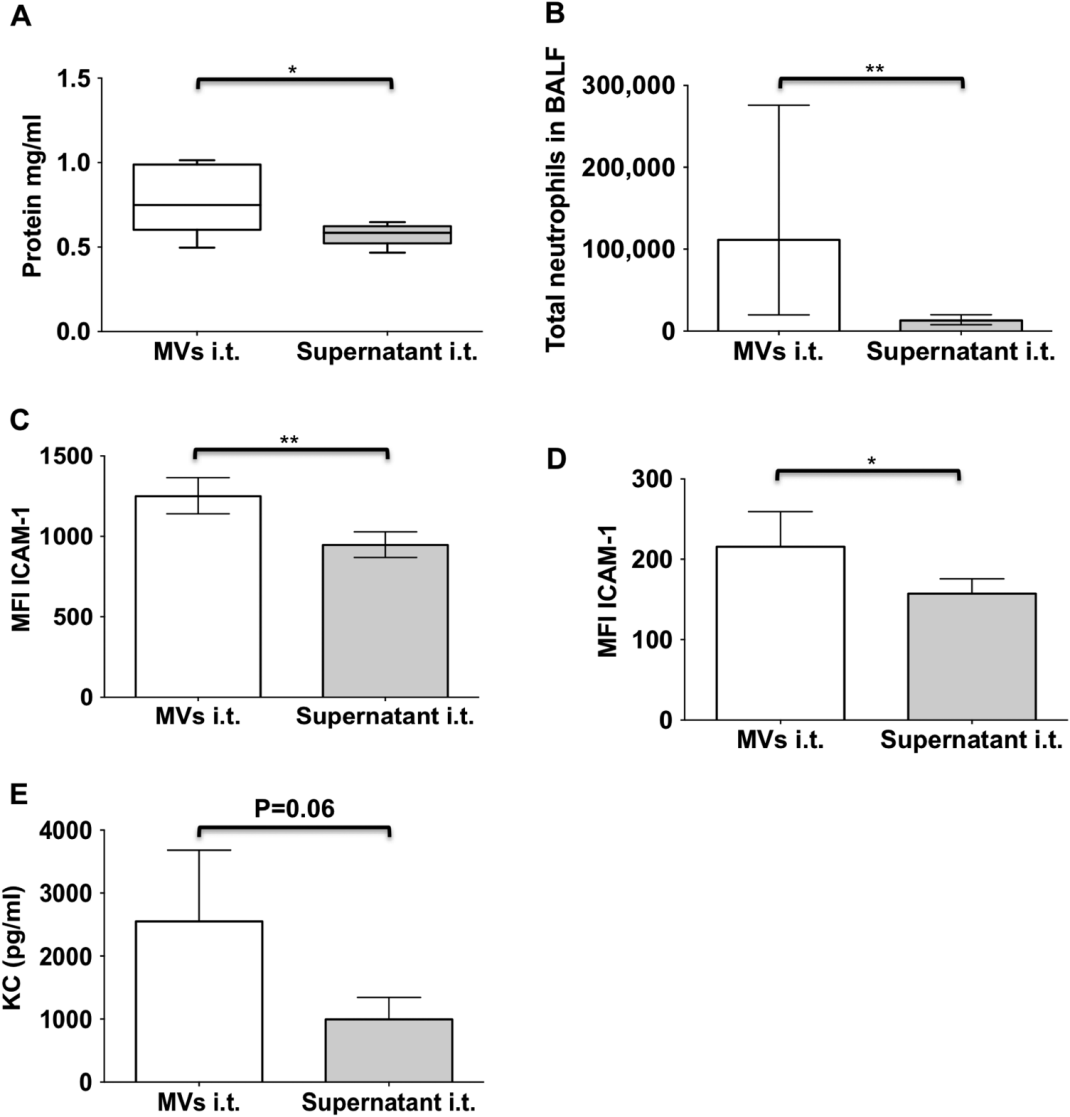
Blinded i.t. instillation of either primary alveolar macrophage-derived inflammatory microvesicles (MVs) or associated supernatant was performed, and multiple parameters of acute lung injury (ALI) were examined. (A) Bronchoalveolar lavage fluid (BALF) protein levels significantly increased following i.t. instillation of MVs, as compared with the supernatant. (B) There was a significant increase in BALF neutrophils 4 hours after instillation of alveolar macrophage-derived MVs. (C) Mice receiving alveolar macrophage-derived MVs also showed a significant increase in surface expression of ICAM-1 on type 1 epithelial cells and (D) type 2 epithelial cells. (E) BALF keratinocyte-derived cytokine (KC) level tended to increase in mice challenged with MVs for 4 hours, but this did not reach statistical significance. N=5, *p<0.05, **p<0.01. Parametric data were analysed by paired t test and displayed as mean+SD (D and E), or back-transformed mean with 95% CIs (lower, upper bounds) for transformed data (B and C). Non-parametric data (A) were analysed by Wilcoxon rank sum test and displayed by box–whisker plots showing the median, IQR and minimum/maximum values. ICAM-1, intercellular adhesion molecule-1.

Having ascertained the proinflammatory effect of these primary alveolar macrophage-derived MVs, we finally assessed their functional activity in vivo. Once again, MVs from LPS-primed alveolar macrophages were subjected to two additional washing steps to minimise any stimulatory effect from remnant soluble factors during the MV-generation process (figure 2). In blinded, paired experiments, mice had either ‘inflammatory’ MVs or post-wash supernatant instilled i.t., and at 4 hours after the challenge, various indices of ALI were examined. As demonstrated in figure 8, MV instillation produced significant increases in lung injury parameters, that is, BALF protein levels and neutrophil number, and surface ICAM-1 expression on both type I and type II epithelial cells. A notable increase in BALF KC was also apparent in mice treated with ‘inflammatory’ MVs, although this was only borderline significant (p=0.06).

## Discussion

This study demonstrates the sequential production of MVs from different precursor cells within the intra-alveolar space early in the pathophysiological process of ALI. Furthermore, we have provided convincing data for a novel role of alveolar macrophage-derived MVs, exerting significant proinflammatory activity mediated by their molecular cargo and potentially contributing to the initiation of ALI in vivo.

We have identified that MVs are readily measureable in BALF in a murine model of ALI. In contrast to previous studies in patients and animals, which have either examined single populations of MV in BALF (eg, epithelial cell MVs^12^ or endothelial cell MVs^14^) or investigated MV release long after ALI/ARDS has been well-established,^12^ ^13^ we found a dynamic production of MVs from multiple intra-alveolar precursor cell populations very early in the inflammatory response. Particularly strikingly, our results demonstrate a substantial increase in MVs originating from alveolar macrophages, which is the first time that alveolar macrophages have been identified as a source of MVs. The kinetics of this response, peaking at around 1 hour before declining, is consistent with their potential early involvement in the inflammatory process of ALI. Epithelial cells were also found to produce substantial numbers of MVs, but with delayed kinetics compared with alveolar macrophages. It has been previously suggested that epithelial cell-derived MVs are upregulated in BALF in patients with ARDS at 6 hours after intubation for respiratory failure,^12^ although these MVs were identified by expression of the receptor for advanced glycation end products (RAGE), and would likely represent a mixture of cell origins as various lung cells express RAGE.^34^ CD11b^+^/Ly6G^+^ MVs were only apparent at 4 hours in our model of LPS-induced ALI, suggesting that these are MVs produced by infiltrating neutrophils. Given the kinetics of these ‘neutrophil-derived’ MVs, it is unlikely that they play a role in initiation of ALI, although they may be a useful biomarker of injury as previously reported.^13^

The pathogenic activity of intra-alveolar MVs remains unknown and led us to investigate their bioactive and functional role. Our data indicate that both a mixed MV population in BALF produced 1 hour after LPS-induced ALI, and a pure alveolar macrophage-derived MV population generated in vitro, activated MLE cells following 4 hours incubation in culture. Furthermore, the administration of alveolar macrophage-derived MVs into naïve mice significantly increased the parameters of ALI tested, clearly demonstrating their capacity as crucial mediators of inflammation in vivo and providing compelling evidence for their role in the pathophysiology of ARDS. It is important to note that these effects were seen just 4 hours after in vivo intratracheal challenge, whereas any functional activity of MVs has previously been reported only after prolonged periods (eg, overnight) of in vitro or in vivo exposure. This highlights a further novelty of this study^14^ ^33^ ^35^ ^36^ and the importance of MVs in the initiation of ARDS.

We have made substantial efforts to address the functional role of MVs in ALI, and as such this study has several strengths compared with other MV research. First, the number of MVs generated from alveolar macrophages (recovered from a single mouse) in vitro was very similar to that found within the BALF of mice 1 hour after LPS instillation (1000–1500/µL) in vivo. It is therefore reasonable to believe that both culture of MLE-12 cells and instillation into mice with these MVs generated in vitro represent an exposure to MVs at physiologically relevant levels. Second, we have used primary cells to generate MVs as opposed to other studies in MV research^14^ ^35–37^ and thus are able to more reliably replicate the phenotypic and proinflammatory activity of endogenous alveolar macrophage MVs. Finally, and crucially, we have used a number of robust controls to highlight the extensive proinflammatory activity of alveolar macrophage-derived MVs. This includes the use of MVs obtained from control untreated mice or non-LPS primed alveolar macrophages, which were seemingly devoid of inflammatory cargo (eg, TNF) and did not induce activation/inflammation in target cells. These data suggest that the proinflammatory activity of MVs is an active process directly mediated by the biological cargo transported within MVs, rather than a passive one based upon MV and target cell contact. Furthermore, while the techniques and reagents required to generate these MVs from primary macrophages may exert some residual stimulating activity, which is the major confounding factor in MV research, this has been carefully excluded in our experiments by the use of post-wash supernatant fraction, obtained from the same MV-generating preparations, as paired controls for the MV treatments. We felt that this is a more pertinent control rather than PBS, as adopted by other published MV research.^14^ ^38^

We found that the proinflammatory role for alveolar macrophage-derived MVs is elicited at least in part via a TNF-dependent mechanism. During LPS-induced ALI, BALF MVs of alveolar macrophage origin packaged TNF on their surface (and potentially inside) in vivo, and anti-TNF treatment was highly effective in abolishing alveolar macrophage MV-induced ICAM-1 upregulation in MLE-12 cells. In contrast, we were unable to detect discernable levels of MV-associated IL-6 or IL-1β early in the pathophysiology of ALI. To our knowledge, this is the first time that cytokine content of intra-alveolar MVs has been investigated. Extracellular vesicles derived from human dendritic cells, predominantly composed of exosomes, have been shown to mediate inflammation via a TNF-dependent pathway.^39^ Our results are consistent with these data and further provide clear identification and quantification of TNF as a bioactive molecular cargo of alveolar macrophage-derived MVs, which has not been previously described. It is important to appreciate here, however, that MVs contain a wide variety of biological mediators including cytokines, chemokines and RNA,^5^ ^6^ ^8^ ^24^ and even lipid fractions of MV membranes themselves have been shown to possess proinflammatory activity.^35^ Thus, it is reasonable to expect that the injurious consequences of MVs generated from alveolar macrophages in vitro and then delivered into mice may be due to a milieu of inflammatory cargo delivered to recipient cells in conjunction with TNF. The key point of our results is the notion that inflammatory cargo carried by MVs (ie, TNF in this study) derived from primed/inflamed alveolar macrophages are indeed able to mediate proinflammatory effects on lung epithelial cells.

There are several caveats to our work. Upon activation, cells also release other species of extracellular vesicles including exosomes, which are smaller in size (30–100 nm) than MVs and also have potential roles in intercellular communication.^4^ Due to the vast differences in MV and exosome biology, it was beyond the scope of this study to investigate the biological/functional roles of exosomes together with MVs. Accordingly, we carefully used differential centrifugation protocols to minimise any contamination of our isolated MV population with exosomes. Our protocols were designed to remove cells and debris (similar protocols resulted in MV populations devoid of apoptotic bodies^35^ ^40^), without introducing exosomes into the MV pellet (exosomes have been reported not to pellet at speed less than 100 000 g^35^ ^40^). It is also relevant to note that due to the limitations of current flow cytometry (particularly machine detection limits and resolution), we are not able to grasp the total population of MVs. In this study, we used a set of conservative yet robust criteria to define MVs, namely, size, parent cell-surface markers and detergent sensitivity, accepting that we may underestimate, but should not overestimate MV numbers and their biological significance. MV numbers within BALF may therefore be even higher than reported here if some of MVs do not express parent cell-surface markers or express them at low levels. Finally, although our results strongly suggest active communication between MVs and target cells, there remain many unanswered questions about the precise nature of this interaction, for example, whether this occurs on the surface of target cells or after MVs’ internalisation. Addressing such fundamental mechanisms would be essential to fully elucidate the role of MVs in the pathophysiology of ALI.

In conclusion, this study demonstrates, for the first time, that MVs from different intra-alveolar precursor cells are sequentially produced within the alveolar space early in the course of ALI. We have clearly shown that these intra-alveolar MVs, principally alveolar macrophage-derived MVs, are potent initiators of inflammation, mediated by their molecular cargo, in particular TNF, and potentially contribute to ALI in vivo. Our results highlight a role for MVs as key components in the pathophysiology of ALI and potentially novel therapeutic targets.
