# Supplementary material for: Alveolar macrophage-derived microvesicles mediate acute lung injury
Source: Thorax. 2016 Jun 10;71(11):1020–9. doi: 10.1136/thoraxjnl-2015-208032 (PMC5099194; doi:10.1136/thoraxjnl-2015-208032)
Supplement: Supplementary data [file thoraxjnl-2015-208032supp.pdf]

ONLINE METHODS SUPPLEMENT

All protocols were approved by the Ethical Review Board of Imperial College London and carried out under the authority of the UK Home Office in accordance with the Animals (Scientific Procedures) Act 1986, UK. 71 Male C57BL/6 mice (Charles River, Margate, UK), aged between 10-14 weeks and weighing between 25-30g, were used. Mice were housed in individual ventilated cages (maximum number of 5 per cage) and exposed to twelve hour light and dark cycles. All experiments were initiated and completed during the light cycle and no adverse effects were observed in any of the treatment groups.

Lipopolysaccharide Model

Randomly selected mice were anesthetized by intraperitoneal injection of ketamine (90mg/kg) and xylazine (10mg/kg). The vocal cords were identified using a microscope and an external light source, a fine catheter was briefly passed 1cm below the cords, and 20µg 'Ultrapure' lipopolysaccharide (LPS) (E. coli O111:B4; InVivoGen, San Diego, CA) in 50µl was instilled intratracheally (i.t.) as previously described[1,2]. The mice were suspended in an upright position for 30 seconds to allow equal bilateral distribution of the LPS. Animals were then placed in a heated box until they had fully recovered from anesthesia. At the end of the experiment, mice were euthanized with anesthetic overdose and exsanguination, an endotracheal tube was inserted via tracheostomy and bronchoalveolar lavage fluid (BALF) was obtained by flushing and gently aspirating 700µl of 0.9% saline in and out of the lungs via the endotracheal tube three times. This was kept on ice and immediately analysed for MV content.

Microvesicle identification

10µL of BALF was incubated with fluorescence-conjugated antibodies against CD11c (clone N418; eBioscience, San Diego CA) and F4/80 (BM8; eBioscience) to identify alveolar macrophage-derived MVs, EpCAM (G8.8; eBioscience) for epithelial cell-derived MVs, or CD11b (M1/70; Biolegend San Diego, CA) and Ly6G (1A8; Biolegend) for neutrophil-derived MVs, for 30 minutes at 4ºC. Samples were re-suspended in 1ml of PBS and then analysed with flow cytometry. MVs were identified as events that were under 1µm in size and positive for specific surface staining markers. Forward scatter, correlating with particle size, and side scatter (trigger threshold 0.02), corresponding with particle granularity, were used to elucidate a 1µm gate that was delineated by specific sizing beads. Absolute MV count was assessed by co-treatment of the sample with a known quantity of 6µm AccuCheck counting beads (PCB100; Life Technologies, Paisley UK). The identity of MVs was validated through their sensitivity to 0.1% Triton X-100 detergent, which solubilizes lipid membranes[3]. Counts of MV populations of different cell origins were compared to untreated wild-type controls. Data was acquired with a Cyan flow cytometer (Beckman Coulter, High Wycombe, UK) and analysis of data was performed with Flowjo software (Tree Star, Ashland, OR).

Cell culture

The murine lung epithelial (MLE-12) cell line was cultured and maintained in 175cm^2 cell culture flasks (Thermo Scientific, Hampshire, UK) in DMEM supplemented with 10% heat inactivated fetal calf serum, penicillin and streptomycin at 37ºC in a humidified 5% CO_2 atmosphere. Cells were seeded at a density of 10^5 cells/well in a 24-well plate (BD falcon, Bedford, MA), maintained in DMEM overnight and then

washed four times in PBS immediately prior to experimentation.

Biological activities of BALF MVs.

TNF, IL-1β and IL-6 content of in vivo-derived MVs was quantified via ELISA kits (R&D Systems, Abingdon, UK). BALF was harvested from mice 1 hour after i.t. LPS or control untreated mice as described above, and centrifuged (200g, 5 mins, 4ºC) to remove cells. The cell-depleted supernatant was then centrifuged at 20,000g for 30mins at 4ºC to sediment MV pellet from MV-depleted BALF. MV pellets were then treated with 1% tween, a mild detergent, to disrupt vesicle membrane and allow extraction of TNF contained within MV. Results were expressed as picograms (pg) within the MV fraction of the sample recovered from BALF taken from a single mouse. MV samples were also incubated with fluorescence-labelled anti-TNF antibody (MP6-XT22; eBioscience) and the quantity of CD11c+ events (MVs originating from alveolar macrophages) that were also positive for TNF was quantified via flow cytometry.

In separate experiments, MVs were isolated from BALF samples, taken from mice 1 hour after i.t. instillation of 20µg LPS or control untreated mice as described above, and washed twice. Washed MVs, or an equivalent volume of post wash supernatant were incubated with murine lung epithelial (MLE-12) cells for 4 hours in the 24-well plate. Following 4 hour challenge, keratinocyte-derived cytokine (KC) in cellular supernatant was determined using ELISA kits (R&D Systems). MLE-12 cells were detached from the plate using trypsin-EDTA (Sigma-Aldrich, Dorset, UK) and incubated with fluorescence-labelled antibody to ICAM-1 (YN1/1.7.4; eBioscience) and its corresponding isotype control, IgG2b κ isotype control (RTK4530; Biolegend), to ascertain surface ICAM-1 expression by flow cytometry as a marker of epithelial

cell activation[4,5]. A variety of other controls were used: PBS; a low dose of LPS (11ng/ml) calculated to be the maximum dose of LPS that may have been retained from the initial instillation within the MV sample despite the washing steps; and a high dose of LPS (100µg/ml), to evaluate responses in the event that MVs carried over LPS.

Biological activity of primary alveolar macrophage-derived MVs

Untreated mice were euthanized (via overdose of anesthetic and exsanguination) and tracheostomy was performed. In order to enhance alveolar macrophage recovery, BALF was harvested with 700µL of calcium-free PBS supplemented with 2mM EDTA, warmed to 37ºC, which was flushed into and gently sucked out of the lungs via the endotracheal tube three times. Each flush was accompanied by gentle massaging of the rib cage to encourage macrophage recovery. Macrophages were isolated by centrifugation (200 g, 5 mins, at 4ºC) and washed 3 times in calcium-containing PBS to neutralize EDTA. They were then placed in 24-well plates and primed for 1hour with 1µg/ml of LPS to attain a pro-inflammatory phenotype as previously described[6] or incubated with PBS alone. Macrophages were then stimulated with 6mM ATP[7] (Tocris, Bristol, UK), 1mM ecto-ATPase inhibitor (ARL67156, Tocris) and 40µM calcium ionophore (A23187, Tocris)[8] for 2 hours to generate either 'inflammatory' or 'non-inflammatory' alveolar macrophage-derived MVs. Stimulation of the purinergic receptor P2X7 results in MV release and ATP plus calcium ionophore synergistically combine to activate P2X7 receptors with resultant MV formation[9]. Whereas ATP alone and ATP plus calcium ionophore generated appreciable levels of alveolar macrophage-derived MVs, the addition of ARL67156 produced much greater quantities (data not shown) of MVs, very similar to numbers

found within the BALF of mice 1 hour after LPS instillation, and hence this combination of stimulants was used to generate MVs from primary alveolar macrophages. Supernatants were subsequently collected and centrifuged to remove cells (200g for 5 mins at 4ºC). Cell-depleted supernatants were then centrifuged at high speed (20,000g for 30 mins at 4ºC) to isolate primary alveolar macrophage-derived MVs.

MVs or the equivalent associated supernatant fraction were incubated with MLE-12 cells for 4 hours and surface ICAM-1 expression was measured by flow cytometry. In separate experiments, MVs were resuspended in 10µg/ml polyclonal anti-TNF antibody (R&D Systems, Abingdon, UK) and then added to MLE cells which had also been pre-treated with 10µg/ml anti-TNF antibody for 10 minutes.

In vitro generated alveolar macrophage-derived MVs initiate ALI in vivo

In vitro generated inflammatory primary alveolar macrophage derived MVs were washed in PBS twice to remove any remaining stimulatory factors. Washed MVs or post-wash supernatant were then instilled i.t. into the lungs of randomly selected mice for 4 hours, by an investigator blinded to the treatment groups. At the end of the protocol mice were euthanized, BALF samples were taken in 700µl 0.9% saline and centrifuged (200g for 5 mins at 4ºC) to separate cells. Using Bradford assay and ELISA kits, BALF Protein (Bio-Rad Laboratories, Hemel Hempstead, UK) and keratinocyte-derived chemokine (KC) levels (R&D Systems) were quantified in cell-depleted BALF.

Lungs were removed and mechanically disrupted in warm fixation buffer using a GentleMACS dissociator (Miltenyi Biotec, Surrey, UK). Samples were then passed through 40µm sieves, washed and resuspended twice in flow cytometry buffer (2%

fetal calf serum, 2mM EDTA and 0.1% sodium azide constituted in PBS) to yield a fixed single cell suspension. Samples were then stained with antibodies at room temperature for 30 minutes to determine ICAM-1 expression on epithelial cells (type 1 epithelial cells were identified as $CD45^-$ (30-F11; Biolegend), $CD31^-$ (MEC 13.3; bdbioscience), $EPCAM^+$ (G8.8; Biolegend) and $T1alpha^+$ (8.1.1; Biolegend) events, whereas type 2 epithelial cells were classified as $CD45^-$, $CD31^-$, $EPCAM^+$ and $T1alpha^-$ as previously described[10] (see additional Figure 1).

Statistical analysis.

Shapiro-Wilk normality tests were carried out and wherever possible, non-parametric data was transformed. Comparisons between two data sets were performed using either paired T-tests or Wilcoxon Rank Sum test. Where three or more datasets were present, ANOVA with Tukey HSD or Kruskal Wallis with Dunn's test were used. Parametric data are presented as mean with SD (untransformed data) or 95% confidence interval (transformed data), whereas non-parametric data are displayed as median with interquartile range. Statistical significance was defined as $p<0.05$ and data were analysed and graphed using IBM SPSS and Prism software.

ADDITIONAL FIGURES

Figure 1

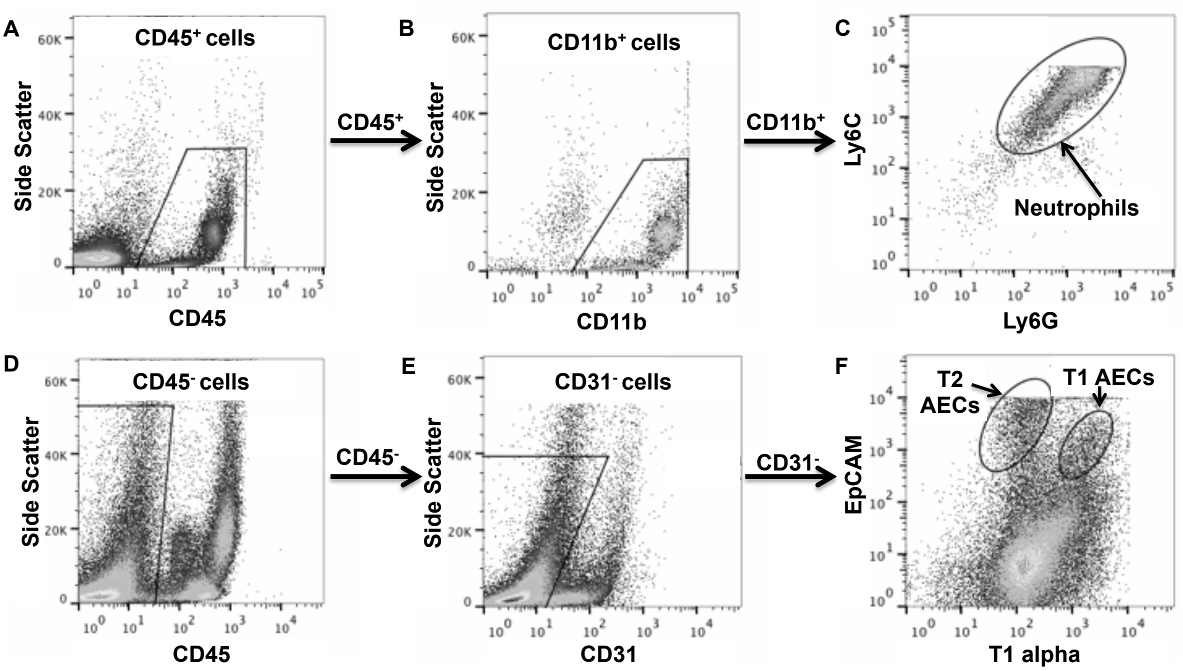

ADDITIONAL FIGURE LEGENDS

Figure 1. Gating strategies for identifying neutrophils in BALF and type 1 and type 2 alveolar epithelial cells (AECs) in lung tissue single cell suspensions by flow cytometry. Neutrophils were identified as $CD45^+$ **(A)**, $CD11b^+$ **(B)**, $Ly6G^+/Ly6C^+$ **(C)**. Type I AECs were identified as $CD45^-$ **(D)**, $CD31^-$ **(E)**, $EpCAM^+/T1\alpha^+$ **(F)** and type II AECs as $CD45^-$, $CD31^-$, $EpCAM^+$, $T1\alpha^-$ **(F)**.

REFERENCE

1. Patel BV, Wilson MR, O'Dea KP, et al. TNF-induced death signaling triggers alveolar epithelial dysfunction in acute lung injury. *The Journal of Immunology* 2013;**190**(8):4274-82.

2. Woods SJ, Waite AA, O'Dea KP, et al. Kinetic profiling of in vivo lung cellular inflammatory responses to mechanical ventilation. *American journal of physiology Lung cellular and molecular physiology* 2015:ajplung. 00048.2015-ajplung. 48.2015.

3. György B, Szabó TG, Turiák L, et al. Improved flow cytometric assessment reveals distinct microvesicle (cell-derived microparticle) signatures in joint diseases. *PLoS One* 2012;**7**(11):e49726.

4. Sebag SC, Bastarache JA, Ware LB. Mechanical Stretch Inhibits Lipopolysaccharide-induced Keratinocyte-derived Chemokine and Tissue Factor Expression While Increasing Procoagulant Activity in Murine Lung Epithelial Cells. *J Biol Chem* 2013;**288**(11):7875-84.

5. Madjdpour C, Oertli B, Ziegler U, et al. Lipopolysaccharide induces functional ICAM-1 expression in rat alveolar epithelial cells in vitro. *American Journal of Physiology-Lung Cellular and Molecular Physiology* 2000;**278**(3):L572-L79.

6. Qu Y, Franchi L, Nunez G, et al. Nonclassical IL-1β secretion stimulated by P2X7 receptors is dependent on inflammasome activation and correlated with exosome release in murine macrophages. *The Journal of Immunology* 2007;**179**(3):1913-25.

7. Rothmeier AS, Marchese P, Petrich BG, et al. Caspase-1-mediated pathway promotes generation of thromboinflammatory microparticles. *J Clin Invest* 2015;**125**(4):1471.

8. Gulinelli S, Salaro E, Vuerich M, et al. IL-18 associates to microvesicles shed from human macrophages by a LPS/TLR-4 independent mechanism in response to P2X receptor stimulation. *European journal of immunology* 2012;**42**(12):3334-45.

9. Thomas LM, Salter RD. Activation of macrophages by P2X7-induced microvesicles from myeloid cells is mediated by phospholipids and is partially dependent on TLR4. *The Journal of Immunology* 2010;**185**(6):3740-49.

10. Woods SJ, Waite AA, O'Dea KP, et al. Kinetic profiling of in vivo lung cellular inflammatory responses to mechanical ventilation. *American Journal of Physiology-Lung Cellular and Molecular Physiology* 2015;**308**(9):L912-L21.
